# Moderate Contrast in the Evaluation of Paintings Is Liked More but Remembered Less than High Contrast

**DOI:** 10.3389/fpsyg.2017.01507

**Published:** 2017-09-05

**Authors:** Katinka Dijkstra, Noah N. N. van Dongen

**Affiliations:** ^1^Department of Psychology, Education, and Child Studies, Erasmus University Rotterdam Rotterdam, Netherlands; ^2^Department of Philosophy, University of Tilburg Tilburg, Netherlands

**Keywords:** art, appreciation, memory, contrast, recognition

## Abstract

Many visual aspects of paintings, as well as exposure to art and cultural norms, contribute to the aesthetic evaluation of paintings. The current study looked at heightened visual contrast as an important factor in the appreciation of paintings. Participants evaluated abstract digitized paintings that were manipulated in contrast for an appreciation task and were later presented with these paintings in a memory task. The results indicated that for art appreciation, a moderate increase in contrast resulted in the highest appreciation for paintings whereas recognition memory was better for paintings with a higher increase in contrast. These results replicate earlier findings with regard to the role of contrast in aesthetic perception and extend these findings by demonstrating a surprising different effect of contrast manipulation for recognition memory. Confidence with which memory decisions were made was in line with art appreciation decisions not memory performance.

## Introduction

When are paintings considered to be works of art? Factors that appear to contribute to these considerations are visual aspects of the object, its perceived similarity to other objects, processing fluency when evaluating it (Reber et al., 2004; Graham et al., 2010), or the hedonic response to the painting (Vessel et al., 2012). As perception of paintings is mainly visual—touch, smell, sound, and taste are usually not part of the experience—and artists are equipped with the same visual and limbic system as the perceiver (Ramachandran and Hirstein, 1999; Zeki, 2001, 2013), visual features of artworks may be important in the evaluation and appreciation of art. Visual contrast is also important because stimuli high in contrast are higher in perceptual fluency than stimuli low in contrast which may affect the appreciation of artworks (Reber et al., 2004; Tinio et al., 2011).

Research examining the role of contrast on the appreciation of visual stimuli and artworks so far has focused on manipulations of different types of contrast and different types of visual stimuli. One study manipulated contrast, sharpness and grain by degrading the stimuli that consisted of photographs of natural and human-made scenes (Tinio et al., 2011). The role of contrast was found to be larger than that of sharpness and grain for the aesthetic appreciation of the stimuli with images degraded in contrast receiving the lowest evaluations. Other studies manipulated visual stimuli in higher or lower figure-ground contrast (Reber et al., 2004). Stimuli higher in figure-ground contrast that were presented briefly were recognized faster and liked better than stimuli lower in this contrast measure and with a longer presentation duration (Reber et al., 1998; Reber and Schwarz, 2001). According to the authors, high processing fluency and aesthetic appreciation of these stimuli go together (Reber et al., 2004), which appears particularly true for stimuli high in contrast. Another study showed that visual stimuli (characters and signs) had higher aesthetic appearance ratings when they had high contrast, such as black-on-white, than when they had low contrast, such as turquoise-on-green, especially a under high luminance contrast condition (Shieh and Lai, 2008). Here, it is the combination of color and luminance contrast that results in high ratings of aesthetic appearance of stimuli that are not associated with art. The results of this study also support the link between aesthetic appreciation of visual stimuli and high contrast levels.

Research with manipulations of contrast in artworks, shows similar results regarding the role of contrast in the appreciation of these artworks. Van Dongen and Zijlmans (2017) manipulated contrast levels in digitized paintings by presenting a lowered luminosity contrast and a heightened luminosity contrast version of a painting to participants. They viewed a pair of paintings with different contrast levels after which they judged which painting they liked better. The results indicated that participants consistently favored high contrast versions over the low contrast counterparts, although the effects of paintings that were high in contrast originally (before the manipulation) were smaller.

Overall, these results form a solid basis for the claim that higher contrast in visual stimuli, including artworks, is appreciated better. Presumably, stimuli with high contrast are processed more easily which in turn evokes a positive affective reaction relative to stimuli with low contrast that are more difficult to process (Winkielman and Cacioppo, 2001). Aesthetic judgments would result from both higher ratings of liking and more positive affect. If high contrast in art forms indeed facilitates processing and contributes to their likeability, two questions arise. The first question is whether increasing contrast results in an ever-higher likeability of this piece of art until the maximum level of contrast is reached. As people recognize high contrast objects more easily (Reber et al., 1998; Reber and Schwarz, 2001), the second question is whether observers would also remember these high contrast art forms better as well. The evidence for a greater likeability of art forms with high contrast (Reber et al., 2004; Shieh and Lai, 2008; Tinio and Leder, 2009a,b; Van Dongen and Zijlmans, 2017) has been discussed above. The answers to the other two questions, however, have not, and form the topic of our investigation.

The first question is what happens under more extreme conditions of contrast compared to the levels of contrast tested before (Van Dongen and Zijlmans, 2017). One outcome could be that appreciation would increase further with increased contrast given the ease of processing in high contrast conditions. The “hedonic fluency model” proposed by Winkielman and Cacioppo (2001) claims that processing fluency of stimuli coincides with a positive affective (hedonic) reaction to them. The results of two empirical studies demonstrated that easier processing was associated with higher positive affect as indicated by changes in emotional reactions on the face (through electromyography) and ratings of self-reported affect. Alternatively, it is feasible that the benefit of processing fluency at a certain level of contrast, diminishes in the sense that at such a *high contrast* level there is no further benefit for processing which results in stimuli being appreciated less than at a *moderate contrast* level. Evidence for this notion is that paintings with initially low contrast were appreciated more when contrast level was manipulated to be higher than for paintings that were already high in contrast initially (Van Dongen and Zijlmans, 2017). This would suggest that art appreciation, as a function of contrast, would asymptotically level off or look like an inverted U-curve with peak sensitivity and peak preferences at an intermediate level (Spehar et al., 2015). Therefore, in the current study, two levels of contrast were manipulated, a *moderate contrast* and a *high contrast* condition, to assess whether further increase in contrast level leads to increased appreciation or not. Given earlier research findings, we hypothesize that it does not.

The second aim of the study is to examine the effect of contrast level on memory for the manipulated works of art that were evaluated earlier. So far, only a few studies have looked at the impact of aesthetically appealing stimuli on memory. One study (Stalinski and Schellenberg, 2013) examined the role of liking novel music excerpts on recognition memory. Participants listened to novel music excerpts and indicated on a 7-point scale how much they liked them. Memory performance was higher for excerpts that had higher likeability rating. This benefit occurred for immediate and delayed memory performance. Apparently, memory for stimuli, and how they are appreciated go hand in hand. This may be the result of a facilitation in processing because of its repeated exposure, also referred to as the “perceptual fluency/attribution model” (Bornstein and D'Agostino, 1994). Because a memory trace for a previously presented item is formed, processing of the item at a later point in time is facilitated. Indeed, in Experiment 6 of the Stalinski and Schellenberg study (2013), in which exposure to the music excerpts was manipulated, an interaction was found between the liking of excerpts with previous exposure, and recognition of these excerpts.

Given these findings on the role of contrast on art appreciation (e.g., Spehar et al., 2015) and the effect of stimuli appreciation on memory performance (Stalinski and Schellenberg, 2013), our hypotheses were as follows. If contrast level is being manipulated into higher contrast levels relative to the original painting, then the *moderate contrast* condition should result in higher appreciation than the *high contrast* condition. The higher appreciation for a *moderate contrast* manipulation in observed paintings should also be present in memory performance, resulting in better recognition memory for paintings appreciated the most in the *moderate contrast* condition.

## Methods

### Participants

A total of 55 participants (67% female, mean age = 26, age range = 19–70) took part in the experiment for course credit. They were mostly 2nd and 3rd year (psychology) students. They participated in the experiment for research credits and were thanked afterwards.

### Stimuli

Stimuli were 100 abstract digitized paintings from digital collections of five established European museums from 1,888 and onward (see Van Dongen and Zijlmans, 2017, for more information). To avoid issues of familiarity, famous or well-known paintings were not included. All stimuli were presented at the width of 500 pixels and with a resolution of 72 dpi. Of the 100 paintings, 80 were used in the first part of the experiment and an additional 20 paintings were used in the second part of the experiment which was the part that tested memory performance.

The paintings were manipulated in two contrast levels relative to the original, normal contrast level, which resulted in three levels total: *original, moderate contrast*, and *high contrast* for each of the 80 paintings for the first part of the experiment. Similar to previous studies (Van Dongen and Zijlmans, 2017), contrast was defined as luminosity contrast and measured by the lightness and amount of pixels, and the range between darkest and lightest pixels. This lightness of pixels was measured on a scale of 256 shades of gray, from black (0) to white (255). For any color, dark shades translate to values from 0 to 127 and light shades translate to values from 128 to 255. Using Adobe Photoshop CS5, contrast was systematically increased by making the dark shade pixels darker, and the light shade pixels lighter. For the *moderate contrast* level, similar to Van Dongen and Zijlmans (2017), the shade of gray 64 and shade of gray 191 were respectively decreased and increased by 15 shades. Toward the neutral values (127 and 128) and the extreme values (0 and 256), changes were progressively smaller with no increase or decrease in the center and the extremes. The same procedure was used for the *high contrast level* except that shade of gray 64 was decreased by 30 shades and shade 191 was increased by 30 shades. No changes were made for the *original contrast level* (see Appendix A in Supplementary Material for an example).

To verify our contrast manipulation, we measured the standard deviation of gray values for all three contrast levels. Based on the amount of gray-valued pixels, the average shade of gray, and the standard deviation in shades of gray can be calculated per painting per contrast level. These standard deviations indicated luminosity contrast; an increase in standard deviation means an increase in the relative amount of pixels near black and white on the gray scale. To test for the increase in contrast between the three levels, we compared their average gray-value standard deviation. Paired-sample *t*-tests showed the expected increase in standard deviation in shades of gray between *normal contrast* and *moderate contrast*, mean difference = 5.78 (*SE* = 0.39), *t*_(78)_ = 14.62, *d* = 1.64, *p* < 0.001 and *moderate contrast* and *high contrast*, mean difference = 8.29 (*SE* = 0.69), *t*_(78)_ = 12.01, *d* = 0.34, *p* < 0.001.

## Procedure

First, participants were presented on the computer with pairs of paintings for which they had to indicate which of the two they preferred (see Appendix B in Supplementary Material for an example). The two paintings were identical except for their level of contrast (see Graham et al., 2016, for a similar procedure). Whether the painting on the right had higher contrast than the painting on the left or not, was randomized. The pairing always consisted of one painting with a normal (*original*) contrast level and one painting with a higher contrast level (*moderate* or *high*). After participants evaluated 80 pairs of paintings, provided demographics information and answered questions about their exposure to art, they commenced with part two of the experiment. In this part, they were presented with half the paintings they had seen before in the appreciation task (old) and 20 new paintings they had not seen before (new). They were instructed to indicate whether or not they had seen the particular painting in the previous part of the experiment with the same level of contrast. In addition, participants were instructed to rate how certain they were of their decision on a seven-point Likert-scale. The memory task consisted of 60 trials; 20 trials with new paintings, 20 trials with paintings the participants saw before with *moderate contrast*, and 20 trials with paintings the participants saw before with *high contrast*. These 40 paintings and their contrast level were randomly selected from the first part of the experiment. On average participants were presented with 20 paintings with contrast levels similar to those of the first part of the experiment (e.g., *moderate contrast—moderate contrast*) and 20 paintings with contrast levels dissimilar to those of the first part of the experiment (e.g., *moderate contrast—high contrast*). As participants had always seen the normal contrast paintings (i.e., the picture they compared the other picture with), *original contrast* paintings were excluded in the memory task. The score on the recognition task was calculated as the percentage correct for previously seen paintings with the same contrast level. After this task, participants answered questions about their eyesight (Do you have normal or corrected to normal eye vision; Do you have any vision defects, such as color blindness) and art experience (How interested are you in art; How knowledgeable are you about art; Are you studying or have you studied art, art history, or a similar subject for the last 2 years). They also had to write down what they thought the goal of the experiment was.

### Design

The design was a within subjects design with three levels of contrast. Dependent variables were appreciation and recognition accuracy. Appreciation was measured as the proportion of preferred increased contrast paintings (*moderate contrast* or *high contrast)* over the *original contrast* versions of the paintings. The procedures followed were in accordance with the ethical standards of the responsible committee on human experimentation.

## Results

Participants were moderately interested in art (*M* = 56.09, *SD* = 28.51 on a scale from 0 to 100) and were not very knowledgeable about art according to their own opinion (*M* = 30.32, *SD* = 20.69 on a scale from 0 to 100). As these variables did not correlate with the dependent variables, they were excluded from further analyses.

**Figure 1:**
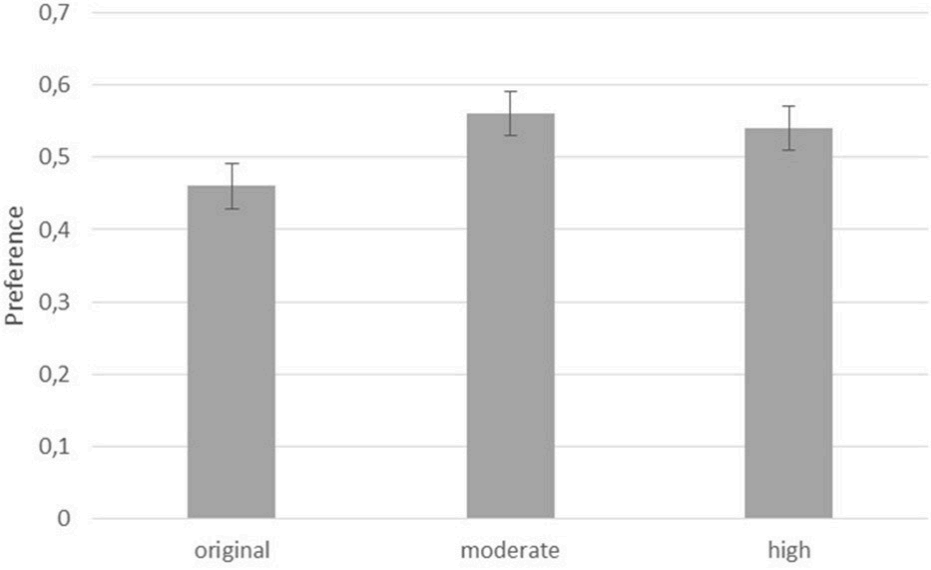
Proportions of preferred paintings per condition.

To assess the effect of contrast on the appreciation of the painting pairs, a 2 (contrast: increased contrast vs. original contrast) by 2 (level of contrast: *moderate contrast* vs. *high contrast)* repeated measures ANOVA was conducted on the choice of appreciation among the participants. The results indicated a main effect of contrast, *F*_(1, 53)_ = 12.76, η^2^ = 0.195, *p* = 0.001, a marginal contrast by contrast level interaction, *F*_(1, 53)_ = 4.02, η^2^ = 0.071, *p* = 0.05. Participants appreciated *moderate contrast* paintings better (*M* = 0.56, *SE* = 0.02) than *original* paintings (*M* = 0.46, *SE* = 0.02). When contrast was manipulated to increase even more, appreciation in the *high contrast* condition (*M* = 0.53, *SE* = 0.01) was lower than in the *moderate contrast* condition, *t*_(53)_ = 2.01, *d* = 0.27, *p* = 0.025, one-tailed. Figure 1 presents the results.

**Figure 2:**
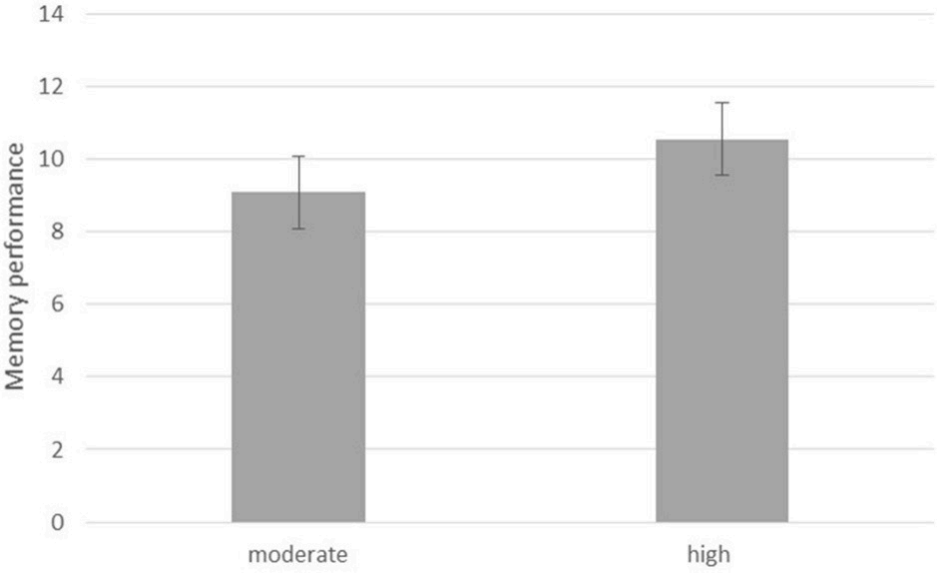
Memory performance for paintings initially shown with moderate and high contrast.

With regard to the memory task, data of four participants were excluded from analysis because of missing data (2) or overly long response times for some trials (2) suggesting that these participants took a break from the experiment which may have affected their memory performance. A paired sample *t*-test was conducted to compare performance on the *moderate* vs. *high contrast* condition as these did not occur for every pair in the appreciation task. There was a significant difference between the two levels of contrast, *t*_(49)_ = 3.18, *d* = 0.45, *p* = 0.003. Participants remembered paintings they saw initially with the *high contrast* better (*M* = 10.54, *SD* = 2.32) than the paintings they saw earlier with the *moderate contrast* (*M* = 9.08, *SD* = 2.32). Figure 2 shows the results.

This result did not support the hypothesis as a similar result as for appreciation was expected. Interestingly, participants were more certain of their performance on the *moderate contrast* (*M* = 5.31, *SD* = 0.62, range = 1–7) than on the *high contrast* paintings they saw earlier (*M* = 5.20, *SD* = 0.62, range = 1–7), *t*_(49)_ = 2.06, *d* = 0.29, *p* = 0.044, even though it did not match their actual performance.

## Discussion

The first hypothesis was that for the manipulation of contrast in the appreciation of paintings, paintings in the *moderate contrast* condition would be appreciated more than paintings in the *high contrast* condition. This hypothesis was supported by the results. Apparently, there is an optimal level of contrast in paintings at which they are being appreciated the most. If contrast is manipulated further, the optimal level of contrast is exceeded and appreciation decreases. This finding supports earlier findings (Spehar et al., 2015) in which further increases in scaling dimensions resulted in lower not higher appreciation of the stimuli. This finding also supports earlier outcomes of a contrast manipulation in which a higher contrast version of paintings were chosen over a lower contrast version (Van Dongen and Zijlmans, 2017). Given the fact that similar paintings and a similar contrast manipulation were chosen (for the *moderate contrast* condition), the results replicate the earlier finding and with this provide more robust evidence for the notion that contrast is an important factor in the appreciation of paintings.

The second hypothesis was that memory for earlier observed paintings with a manipulated contrast level would be highest for the paintings in the *moderate contrast* condition. This turned out not to be the case. Instead, paintings initially observed in the *high contrast* condition were remembered best. This result did not support the hypothesis nor earlier findings that indicated better memory performance stimuli that were appreciated more and had a processing benefit of previous exposure (Stalinski and Schellenberg, 2013). Apparently, the mechanisms underlying appreciation for contrast-manipulated art and the one underlying memory for contrast-manipulated art are not the same.

An explanation could be that the *high level of contrast* facilitated processing and subsequent memory of the stimuli because they were more distinctive due to the higher contrast level. Even though the paintings in the *moderate contrast* condition were appreciated the most, appreciation of the paintings in the *high contrast* condition was still higher than the appreciation of the original contrast level. Interestingly, confidence levels for the memory task were higher for the *moderate* than the *high* contrast condition. According to the perceptual fluency/attribution model account, participants tend to attribute fluency to the observed stimulus when memory performance is high to a greater extent than to the earlier affective response of the stimulus (Bornstein and D'Agostino, 1994). This suggests that memory performance may have relied more on the distinct feature of the actual visual source (*high contrast*) whereas the confidence regarding the memory decision may have relied less on the visual feature of the source but more on the appreciation associated with it, i.e., the earlier affective response associated. The relative low and non-significant correlations between performance and confidence levels (*r* < 0.260) support this notion.

If this were true, and this should be corroborrated by other, independent studies, then this would provide deeper insight into the process of and opportunities for learning. Manipulating visual stimuli could have differential effects on affective responses, recognition memory, and subjective reports of confidence level with which decisions regarding previously observed stimuli are being evaluated. This can be taken into account when employing different tasks regarding art appreciation and memory for art stimuli.

Overall, this study provides a deeper understanding of the effects of contrast manipulations of paintings on their appreciation and memory. Formal characteristics and manipulations of art seem to have an impact that goes beyond perceptual processing and basic observation. This has not only been demonstrated with regard to art (Krentz and Earl, 2013; Van Dongen and Zijlmans, 2017) but with regard to reading comprehension as well (Hoeben Mannaert et al., 2017). Generally, manipulations to formal characteristics of modality-specific stimuli appear to have a major effect on either their processing, appreciation, memory or a combination of these elements.

Implications of this study are that there may be a limit as to how far a manipulation can be extended for it to have an effect (see also Hoeben Mannaert et al., 2017) and that factors considered to be converging around a manipulation, such as appreciation, memory, and confidence level on a decision task, may not be as convergent as predicited. Future studies should focus more on the potential independence of these factors as they may yield relevant insights into the mechanisms underlying art perception, art appreciation and memory for works of art.

## Author contributions

KD and Nv designed the study and research question together and supervised the data collection. Noah was responsible for the materials, design, and data analysis. KD wrote the paper.

### Conflict of interest statement

The authors declare that the research was conducted in the absence of any commercial or financial relationships that could be construed as a potential conflict of interest.
