# Supplementary material for: Moderate Contrast in the Evaluation of Paintings Is Liked More but Remembered Less than High Contrast
Source: Front Psychol. 2017 Sep 5;8:1507. doi: 10.3389/fpsyg.2017.01507 (PMC5591943; doi:10.3389/fpsyg.2017.01507)
Supplement: Supplementary file 1 [file DataSheet1.docx]

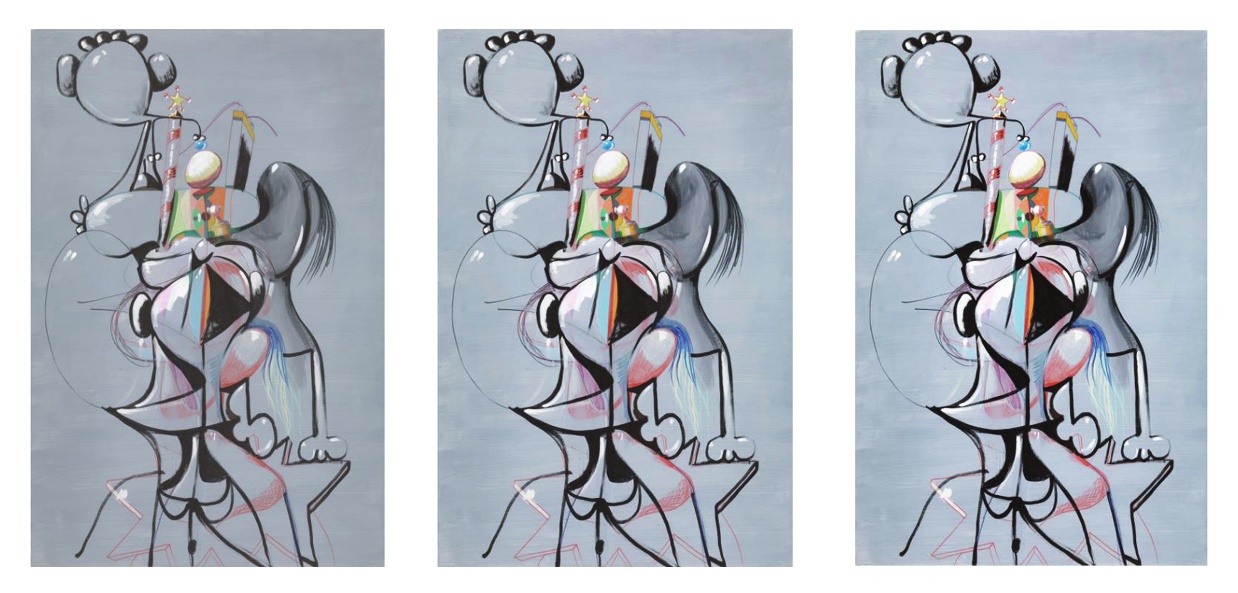
Appendix A

Three levels of contrast of the painting *Colored Dream Objects* by George Condo from the online collection of the Tate Gallery (London, UK). From left to right: *normal contrast, moderate contrast, high contrast.*


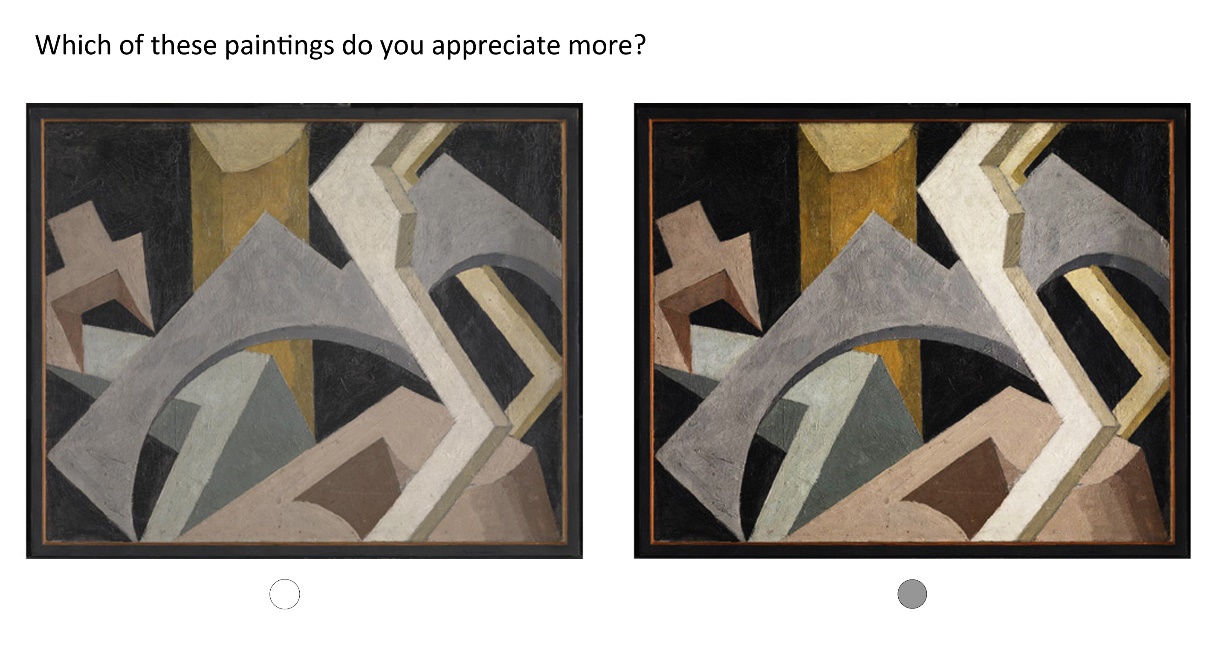
Appendix B

Stimulus example of the first part of the experiment.
